# The Neighborhood Energy Balance Equation: Does Neighborhood Food Retail Environment + Physical Activity Environment = Obesity? The CARDIA Study

**DOI:** 10.1371/journal.pone.0085141

**Published:** 2013-12-27

**Authors:** Janne Boone-Heinonen, Ana V. Diez-Roux, David C. Goff, Catherine M. Loria, Catarina I. Kiefe, Barry M. Popkin, Penny Gordon-Larsen

**Affiliations:** 1 Department of Public Health and Preventive Medicine, Oregon Health and Science University, Portland, Oregon, United States of America; 2 Department of Epidemiology, University of Michigan, Ann Arbor, Michigan, United States of America; 3 Department of Epidemiology and Prevention, Wake Forest University School of Medicine, Winston-Salem, North Carolina, United States of America; 4 Division of Epidemiology and Clinical Applications, National Heart, Lung, and Blood Institute, Bethesda, Maryland, United States of America; 5 Department of Quantitative Health Sciences, University of Massachusetts Medical School, Worchester, Massachusetts, United States of America; 6 Department of Nutrition, Carolina Population Center, University of North Carolina at Chapel Hill, Chapel Hill, North Carolina, United States of America; Old Dominion University, United States of America

## Abstract

**Background:**

Recent obesity prevention initiatives focus on healthy neighborhood design, but most research examines neighborhood food retail and physical activity (PA) environments in isolation. We estimated joint, interactive, and cumulative impacts of neighborhood food retail and PA environment characteristics on body mass index (BMI) throughout early adulthood.

**Methods and Findings:**

We used cohort data from the Coronary Artery Risk Development in Young Adults (CARDIA) Study [n=4,092; Year 7 (24-42 years, 1992-1993) followed over 5 exams through Year 25 (2010-2011); 12,921 person-exam observations], with linked time-varying geographic information system-derived neighborhood environment measures. Using regression with fixed effects for individuals, we modeled time-lagged BMI as a function of food and PA resource density (counts per population) and neighborhood development intensity (a composite density score). We controlled for neighborhood poverty, individual-level sociodemographics, and BMI in the prior exam; and included significant interactions between neighborhood measures and by sex. Using model coefficients, we simulated BMI reductions in response to single and combined neighborhood improvements. Simulated increase in supermarket density (from 25^th^ to 75^th^ percentile) predicted inter-exam reduction in BMI of 0.09 kg/m^2^ [estimate (95% CI): -0.09 (-0.16, -0.02)]. Increasing commercial PA facility density predicted BMI reductions up to 0.22 kg/m^2^ in men, with variation across other neighborhood features [estimate (95% CI) range: -0.14 (-0.29, 0.01) to -0.22 (-0.37, -0.08)]. Simultaneous increases in supermarket and commercial PA facility density predicted inter-exam BMI reductions up to 0.31 kg/m^2^ in men [estimate (95% CI) range: -0.23 (-0.39, -0.06) to -0.31 (-0.47, -0.15)] but not women. Reduced fast food restaurant and convenience store density and increased public PA facility density and neighborhood development intensity did not predict reductions in BMI.

**Conclusions:**

Findings suggest that improvements in neighborhood food retail or PA environments may accumulate to reduce BMI, but some neighborhood changes may be less beneficial to women.

## Introduction

Policies to combat obesity have increasingly considered neighborhood modifications to improve access to healthy food options and places to be physically active. Policies draw from two largely distinct literatures, mostly from specialists in diet or physical activity. On the physical activity side, research has focused on the availability of neighborhood physical activity facilities [1–3] and community designs in which residential, commercial, and other infrastructure are abundant [4,5] (development intensity) and intermixed (mixed land use) [6–8]. Parallel research on the food retail environment typically focuses on increasing the availability of affordable, fresh produce through supermarkets or reducing access to energy dense, nutrient poor foods available in fast food restaurants and convenience stores [9–16]. However, few studies have data on both food retail and physical activity neighborhood exposures required for comprehensive examinations of the obesogenic environment. 

There is little understanding of which aspects of the neighborhood environment are comparatively stronger in predicting obesity, and thus offer greatest policy potential. Nor is there understanding of how elements of neighborhood food retail and physical activity environments might interact to reduce obesity. While a growing number of studies [17–25] examine neighborhood food retail and physical activity environments in relation to health outcomes, none have estimated their interactive effects. In addition, few studies examine differential neighborhood effects according to gender, and with notable exceptions [5,9–11,26–30], the predominance of the literature is derived from small study populations using cross-sectional study designs. 

Using 18 years of longitudinal clinical data and geographically- and temporally-linked Geographic Information Systems-derived neighborhood measures in a large, biracial cohort, we first estimated the joint and interactive impacts of neighborhood food retail and physical activity environment features on body mass index (BMI) throughout early adulthood. Second, we estimated between-exam reductions in BMI predicted from single improvements to neighborhood food retail and physical activity environments. Third, we estimated cumulative effects of multiple neighborhood improvements on BMI, focusing on the most promising policy targets from the second step.

## Methods

### Study Population and Data Sources

The Coronary Artery Risk Development in Young Adults (CARDIA) Study is a community-based prospective study of the determinants and evolution of cardiovascular risk factors among young adults. At baseline (1985-6), 5,115 eligible subjects, aged 18-30 years, were enrolled with balance according to race (African American and white), gender, education (≤ and high school) and age (18-24 and 25-30 years) from the populations of Birmingham, AL; Chicago, IL; Minneapolis, MN; and Oakland, CA. Specific recruitment procedures were described elsewhere [31]. Written consent and study data were collected under protocols approved by Institutional Review Boards at each study center: University of Alabama at Birmingham, Northwestern University, University of Minnesota, and Kaiser Permanente. Geographic linkage and analysis for the current study was approved by the Institutional Review Board at the University of North Carolina at Chapel Hill. Follow-up examinations conducted in 1987-1988 (Year 2), 1990-1991 (Year 5), 1992-1993 (year 7), 1995-1996 (year 10), 2000-2001 (year 15), 2005-2006 (year 20), and 2010-2011 (year 25) had retention rates of 90%, 86%, 81%, 79%, 74%, 72%, and 72% of the surviving cohort, respectively. 

Using a Geographic Information System, we linked time-varying, neighborhood-level food resource and United States (U.S.) Census data to CARDIA respondent residential locations in exam years 0, 7, 10, 15, and 20 from geocoded home addresses (year-specific street segment matches achieved for 92.5, 94.0, 90.7, 93.3, and 93.0% of respondents, respectively). Among participants at baseline, 48.7, 70.8, 36.7, and 51.8% moved residential locations between years 0 and 7, 7 and 10, and 10 and 15, and 15 and 20, respectively. 

In our study, we used a full longitudinal model for the entire 18 year period to examine neighborhood measures from years 7, 10, 15, and 20 in relation to BMI from years 10, 15, 20, and 25, respectively, controlling for covariates concurrent with neighborhood measures. The rationale for our lag time of 3-5 years was to capture a period in which a meaningful change in BMI could occur (average 5-year weight gain in the CARDIA study was approximately 1.5 and 1.1 BMI units in blacks and whites, respectively [32]). We omitted Year 0 data due to inconsistent coding of fast food restaurants and food stores in Dun and Bradstreet data corresponding to Year 0 (1985).

Of 15,254 person-exam observations in which participants attended Year 7, 10, 15, or 20 exams, we excluded person-exam observations in which women were pregnant (n=166) or had missing height or weight (n=2,041) in concurrent or time-lagged exams. We also excluded person-exam observations with missing covariate data (n=125 additional exclusions) in years concurrent with neighborhood measures (Years 7, 10, 15, 20). Neighborhood environment data were nearly complete (n=1 additional exclusion) for all person-exam observations, so exclusion was unrelated to the study exposures. Additionally, our fixed effects models may mitigate selection bias (attrition and missing data) related to unobserved fixed individual-level characteristics. Our final analytic sample included 12,921 person-exam observations representing 4,092 unique individuals.

### Neighborhood environment measures

Neighborhood food retail and physical activity resources were obtained from Dun and Bradstreet, a commercial dataset of U.S. businesses [33]. In this study, we focused on neighborhood features that (1) are relevant for current policy changes or (2) have empirical evidence for longitudinal association with diet, physical activity, or BMI: fast-food chain restaurants, supermarkets (large grocery stores), convenience stores, commercial physical activity facilities, public physical activity facilities, and facilities supporting sedentary activities (e.g., movie theaters, arcades) corresponding to each CARDIA exam period were extracted and classified according to 8-digit Standard Industrial Classification codes [34] (**Tables S1-S3 **in File **S1**). Accelerated increase in resource density from Year 15 to 20 (Table **2**) likely reflects coding changes; compared to changes in resource density from Year 10 to 15, changes in resource density from Year 15 to 20 were unrelated to race, sex, and education (data not shown). Based on prior work [10,35], counts of each type of resource were calculated within 3 km of each respondent’s residential location (Euclidean buffers). Resource availability and population density are independently related to behavior [36–38]; to estimate their independent effects, we examined population-scaled measures of resource density (number of resources per 10,000 or 100,000 population, depending upon frequency of the given resource), which help to separate resource availability from population density. Population counts were derived from U.S. Census block group [39] population counts, weighted according to the proportion of block-group area within the 3 km neighborhood buffer. 

**Table 2:** Neighborhood-level descriptive characteristics of residential neighborhoods at baselines and changes over time^a^ [median or median change (10^th^, 90^th^ percentile)].

	Median	Median Change		
	Year 7	Year 10 – Year 7	Year 15 – Year 10	Year 20 – Year 15
Fast food restaurants^b^	0.9 (0.4, 2.2)	0.1 (-1.0, 1.4)	0.0 (-1.1, 1.0)	0.8 (-0.4, 3.6)
Supermarkets^c^	4.0 (0.0, 11.0)	-0.1 (-5.5, 5.4)	0.0 (-5.1, 4.8)	2.7 (-2.6, 10.6)
Convenience stores^b^	4.7 (3.1, 7.7)	-0.7 (-3.2, 3.3)	-0.1 (-2.1, 2.4)	0.7 (-1.8, 4.3)
Commercial physical activity facilities^b^	1.8 (0.5, 4.4)	0.4 (-1.5, 3.0)	0.5 (-1.3, 3.2)	1.7 (-0.6, 6.3)
Public physical activity facilities^b^	0.4 (0.0, 1.0)	0.0 (-0.6, 0.6)	0.0 (-0.5, 0.6)	0.1 (-0.4, 0.9)
Development intensity^d^	-0.1 (-0.6, 1.3)	-0.1 (-1.2, 0.2)	0.0 (-0.2, 0.2)	0.0 (-0.3, 0.2)
Neighborhood-level poverty^e^	0.2 (0.1, 0.5)	0.0 (-0.3, 0.1)	0.0 (-0.1, 0.1)	0.0 (-0.1, 0.1)

^a^ Coronary Artery Risk Development in Young Adults (CARDIA) Study, 1992-2011

^b^ Resource density (counts per 10,000 population) within 3km Euclidean buffer

^c^ Resource density (counts per 100,000 population) within 3km Euclidean buffer

^d^ Development intensity score constructed from population density (1990 and 2000 U.S. Census for CARDIA years 7 & 10 and 15 & 20, respectively), road density, and total resource (all food, physical activity, and inactivity facilities) using Exploratory Factor Analysis

^e^ Proportion households <150% of poverty within census tract (1990 and 2000 U.S. Census for CARDIA years 7 & 10 and 15 & 20, respectively)

We characterized development intensity using exploratory factor analysis of street connectivity (link to node ratio) and population density, road density, and total resource density (all food retail, physical activity, and inactivity resources of any type) per square kilometer within 1 km Euclidean buffers [35] in data pooled across years. Link to node ratio and road miles were extracted from ESRI Streetmap data (2000 [40], 2005 [41], and 2010 [42] for years 7 & 10, 15, and 20, respectively). After excluding link to node ratio [factor loadings (<0.05), uniqueness (0.998)], a single factor represented the remaining three variables (Eigenvalue=1.28, Table **S4** in File **S1**). Development intensity within 3 km yielded similar results (**Tables S4 and S5 **in File **S1**). 

 Neighborhood poverty was defined as percent of persons <150% of federal poverty level (1.5*federal poverty level [43]) within the respondent’s census tract of residence, derived from 1990 and 2000 U.S. Census data matched to CARDIA exam years 7 & 10 and 15 & 20, respectively. 

### Body Mass Index (BMI; outcome variable)

Weight and height were measured according to standardized protocol described previously [44]. BMI was calculated as weight (kg) / height (m)^2^ at each exam. 

### Control variables

Individual-level baseline characteristics included race (white, black), and study center. Highest education reported (≤high school, some college, college graduate) across Years 0, 5, 7, 10, 15, 20, and 25 was examined as a time-constant variable. Time-varying individual-level characteristics included age (in years), income (in 10,000 U.S. dollars), marital status (married, not married), children or stepchildren ≤18 years living in the household (any, none), and tobacco use (current, not current). Income was inflated to 2001 U.S. dollars using the Consumer Price Index [45]. Missing income (n=116 observations; 0.9% among 12,921 in final sample) was imputed based on individual-level age, race, sex, education, and study center; and residence within or outside of an urbanized area, census tract-level median household income, and county-level cost of living index. 

### Statistical Analysis

#### Step 1: Longitudinal regression model

Effects of neighborhood food retail and activity resources and development intensity (time t) on BMI in the next time period (time t+1) throughout young to middle adulthood were estimated in a fixed effects longitudinal model. The fixed effects model exploits the repeated measures of neighborhood environment and BMI in the CARDIA study by conditioning on each individual, thereby analyzing variation observed within person, over time. In this way, fixed effect models control for time-constant unmeasured variables (e.g., diet behaviors that remain constant over time) [29,46,47]; in essence, each individual served as his/her own control. Models controlled for time-varying age, income, marital status, children, tobacco use, and neighborhood poverty at time t; because fixed effects models rely on within-person variation, coefficients for time-constant variables (study center, education, race, sex) were not estimated. We also controlled for BMI at time t in order to adjust for other factors that determined BMI prior to the inter-exam neighborhood changes analyzed in our longitudinal models. 

The Hausman specification test [48] indicated significant confounding by time-constant unmeasured variables (p<0.001) and, therefore, that fixed effects modeling was more appropriate than random effects models (random person-level intercept) which analyze variation both within and between individuals. Models were fit using Stata 10.1 longitudinal regression (xtreg, using the “fe” option) [49]. As described elsewhere [47], we treat neighborhood poverty as an individual-level exposure. Consistent with prior work [10,29], we used the natural-log transformation of neighborhood food retail and physical activity resource variables to linearize the relationships and reduce the influence of right-skewed resource counts on model estimates.

We tested interactions among sex, neighborhood poverty, and built environment characteristics in three cumulative steps. First, because relationships between neighborhood food retail and physical activity environment characteristics and diet and physical activity vary according to sex [10,29], we tested and retained significant (Wald p<0.10) interactions between neighborhood measures and sex. Second, neighborhood food retail and physical environment characteristics may affect obesity-related outcomes differently in socioeconomically advantaged and disadvantaged neighborhoods, due to unmeasured differences in resources, behavior patterns, and access barriers. Therefore, we tested and retained pairwise interactions between built environment measures and neighborhood poverty. Third, to explore how different aspects of neighborhood food retail and physical activity environments operate together, we tested all single pairwise interactions between each food retail and physical activity environment measure. We then combined significant interactions into a cumulative model that contained covariates and sex/neighborhood poverty interactions, then removed interactions that were no longer significant. 

#### Step 2: Calculate change in BMI expected from neighborhood improvements

To simulate policy changes, we used estimated coefficients from the Step 1 model to calculate changes in BMI expected from changes in neighborhood measures. We present findings contrasting the 25^th^ and 75^th^ percentiles (in the pooled sample) of single and multiple neighborhood measures. In our simulation of multiple neighborhood improvements, we focused on promising policy targets, defined as modifiable neighborhood measures that were associated with BMI in the direction consistent with recent policy strategies (BMI positively associated with fast food restaurant and convenience store density with BMI, and negatively associated with development intensity and supermarket and physical activity facility density). Thus, in our estimation of cumulative effects of multiple neighborhood changes, we did not include neighborhood measures that were unrelated to BMI or related to BMI in the unexpected direction. Simulations incorporated statistically significant interactions included in the Step 1 model. 

## Results

Compared to men, a greater proportion of women were black, had higher education and lower income, had higher BMI, and lived with children; a smaller proportion smoked (Table **1**). Men and women did not differ according to age or marital status. Neighborhood characteristics exhibited substantial variation across individuals; for example, while the median supermarket density at year 7 was 4.0 markets per 100,000 population within 3 km of each residence, the 10^th^ and 90^th^ percentiles were 0 and 11 per 100,000 population, respectively. Within-person temporal variation in neighborhood measures was comparable or larger in magnitude than between-person differences at Year 7 (Table **2**). 

**Table 1:** Individual-level sample characteristics, by sex [mean/% (standard error)] ^a^.

		Men	Women
		(n=1,810)	(n=2,282)
White^*^ (%)		50.9	49.1
Education^b*^ (%)	≤HS	36.3	30.5
	Some college	17.6	21.1
	≥College grad	46.1	48.3
Married^c^ (%)		45.6	44.1
Child(ren) in household^cd*^ (%)		36.1	51.1
Current smoker^c*^		28.1	24.4
Age^c^ (mean)		32.1 (0.1)	32.1 (0.1)
Income, in $10,000^ce*^ (mean)		5.6 (0.1)	5.2 (0.1)
Body Mass Index^*^ at Year 7 (mean)		26.5 (0.1)	26.9 (0.2)
Body Mass Index^*^ at Year 20 (mean)		29.3 (0.2)	30.3 (0.2)

^a^ Coronary Artery Risk Development in Young Adults (CARDIA) Study, 1992-2011

^b^ Highest education reported through Year 20

^c^ At baseline (Year 7)

^d^ Children or stepchildren <18 years living in household

^e^ Inflated to reflect value of 2000 U.S. dollars

^*^ Significant difference between men and women (p<0.05) per t-test or Pearson chi-square

### Independent and interactive relationships among neighborhood measures

In the multivariable fixed effects model, a 10% increase in supermarket density was associated with small decrease in BMI (0.009 kg/m^2^; coefficient = -0.09; Table **S5** in File **S1**). Density of fast food restaurants and convenience stores and development intensity were not associated with BMI. Public physical activity facilities exhibited positive interaction with neighborhood poverty and negative interaction with commercial physical activity facilities (interaction p<0.10). In addition, a three-way interaction among commercial public facilities, sex, and neighborhood poverty was significant. Crude models are reported in Table **S6** in File **S1** **.**

While our model contains many variables and interactions, neighborhood variables were only moderately correlated (Spearman r<0.5; see Table **S7** in File **S1**). We observed at least 400 person-time observations in 25^th^ and 75^th^ percentile cross-classifications of each pair of neighborhood characteristics (data not shown). To facilitate interpretation of these complex models, we conducted simulations that contrast the 25^th^ and 75^th^ percentiles of each neighborhood characteristic.

### Predicted changes in BMI with changes to single elements of food retail or physical activity environments

In a series of simulations, we estimated the impact of single changes to the neighborhood food retail or physical activity environment (e.g., reducing fast food restaurant density) (Table **3**), accounting for the numerous interactions included in our final model. Increases in neighborhood supermarket density from the 25^th^ percentile (1.2/100,000 population) to the 75^th^ percentile (2.2/100,000 population) predicted a mean 0.09 kg/m^2^ reduction in BMI in a period roughly equivalent to time between CARDIA exams (approximately 5 years). Reducing fast food restaurant and convenience store density and increasing neighborhood development intensity predicted small, non-statistically significant changes in BMI. 

**Table 3:** Predicted change in BMI with changes to single elements of the neighborhood environment^**a**^.

Simulated change [From, To]^b^	Subgroup^c^	Predicted BMI Change (95% CI)
Reduce fast food restaurant density (fast food)^d^ [1.10, 0.51]		0.03 (-0.05, 0.11)
Increase supermarket density (supermarket)^e^ [1.18, 2.22]		-0.09 (-0.16, -0.02)*
Reduce convenience store density (convenience)^d^ [1.97, 1.44]		-0.02 (-0.09, 0.05)
Public physical activity facility density (public)^d^ [0, 0.60]	Low^b^ neighborhood poverty, Low commercial facilities	-0.01 (-0.17, 0.15)
	Low^b^ neighborhood poverty, High commercial facilities	-0.09 (-0.22, 0.04)
	High^b^ neighborhood poverty, Low commercial facilities	0.22 (0.06, 0.37)*
	High^b^ neighborhood poverty, High commercial facilities	0.14 (0.00, 0.29)*
Commercial physical activity facility density^d^ [0.91, 1.74]	Men	
	Low^b^ neighborhood poverty, Low public facilities	-0.15 (-0.29, -0.01)*
	Low^b^ neighborhood poverty, High public facilities	-0.22 (-0.37, -0.08)*
	High^b^ neighborhood poverty, Low public facilities	-0.14 (-0.29, 0.01)
	High^b^ neighborhood poverty, High public facilities	-0.21 (-0.36, -0.06)*
	Women	
	Low^b^ neighborhood poverty, Low public facilities	0.02 (-0.12, 0.15)
	Low^b^ neighborhood poverty, High public facilities	-0.06 (-0.20, 0.08)
	High^b^ neighborhood poverty, Low public facilities	0.13 (-0.02, 0.28)
	High^b^ neighborhood poverty, High public facilities	0.06 (-0.09, 0.21)
Increase development intensity [-0.50, 0.20]		-0.02 (-0.08, 0.04)

^a^ Estimated using fixed effects linear regression modeling Body Mass Index (BMI, kg/m^2^) as a function of fast food restaurant, convenience store, supermarket, commercial physical activity facility, and public physical activity facility density within 3km buffers and development intensity within 1km buffers (Euclidean buffers around each respondent’s residential location), and proportion of persons below 150% of federal poverty level; Coronary Artery Risk Development in Young Adults (CARDIA) Study (1992-2011). The fixed effects model is adjusted for time-varying income, marital status, children in household, and significant (p<0.10) interactions between neighborhood measure and gender, and significant pairwise interactions among neighborhood measures; race, education, and study center are time invariant and therefore omitted from fixed effects models. Predictions apply estimated coefficients from final fixed effects model (Table S5 in File **S1**; n=12,921 person-exam observations representing 4,092 individuals).

^b^ Corresponds with 25^th^ and 75^th^ percentiles.

^c^ BMI change predicted for simulated neighborhood changes within subgroups defined by neighborhood measures with significant interactions^a^

^d^ Resource density (counts per 10,000 population) within 3km Euclidean buffer

^e^ Resource density (counts per 100,000 population) within 3km Euclidean buffer

^*^ p<0.05

Increases in public facilities predicted significant *increases* in BMI in high poverty neighborhoods, with some variation according to density of commercial physical activity facilities; public facility density was unrelated to BMI in low poverty neighborhoods (Table **3**). Increasing the density of neighborhood commercial physical activity facilities predicted reductions (ranging from 0.14 to 0.22 kg/m^2^) in BMI only in men, which were greater in areas with high density of public physical activity facilities. 

### Predicted changes in BMI with combined neighborhood environment changes

**Figure 1:**
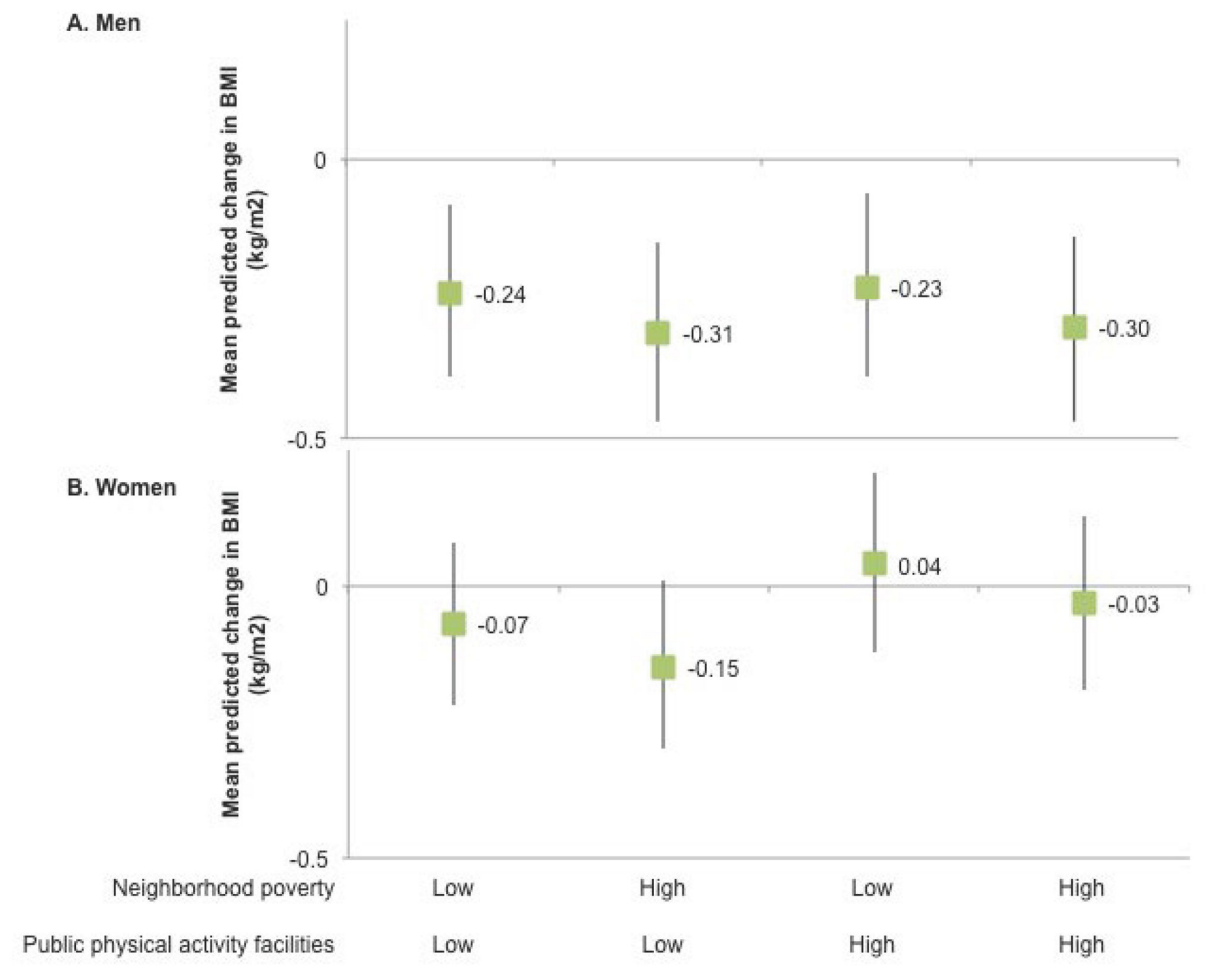
Multi-component policy change simulation: predicted change in BMI^ab^. ^a^Predicted change in BMI with increased availability of supermarkets and commercial physical activity facilities, by neighborhood poverty and availability of commercial physical activity facilities. Estimated using fixed effects linear regression modeling Body Mass Index (BMI, kg/m^2^) as a function of fast food restaurant, convenience store, supermarket, commercial physical activity facility, and public physical activity facility density within 3km buffers and development intensity within 1km buffers (Euclidean buffers around each respondent’s residential location); Coronary Artery Risk Development in Young Adults (CARDIA) Study (1992-2011). The fixed effects model is adjusted for time-varying age, income, marital status, children in household and proportion of persons below 150% of federal poverty level and significant (p<0.10) interactions between neighborhood measure and gender, and significant pairwise interactions among neighborhood measures; race, education, and study center are time invariant and therefore omitted from fixed effects models. Predictions apply estimated coefficients from final fixed effects model (Table **S5** in File **S1**; n=12,921 person-exam observations representing 4,092 individuals). Error bars represent 95% confidence intervals. ^b^Resource density is calculated as counts per 10,000 population within 3km Euclidean buffer. “High” and “low” levels correspond to 25^th^ and 75^th^ percentiles for each measure among all pooled person-exam observations.

To simulate effects of combined changes to the neighborhood environment, we focused on two neighborhood policy targets that were associated with BMI in the direction assumed by recent policies: increasing the density of neighborhood supermarkets and commercial physical activity facilities, each from the 25^th^ to 75^th^ percentile of the distribution. Compared to changes in single elements of neighborhood food retail or physical activity environments, increases in both supermarket and commercial physical activity facilities predicted larger and more consistent reductions in BMI ranging from 0.23 to 0.31 kg/m^2^ in men (Figure **1**). For example, we predicted a mean decrease in BMI of 0.31 kg/m^2^ resulting from increased supermarket and commercial physical activity facility density in areas with high poverty and low public physical activity facility density. While the statistical interactions retained in the model were statistically significant, the predicted changes in BMI were not significantly different from each other (Wald test, p<0.05; data not shown). Among women, combined neighborhood changes predicted no significant changes in BMI.

## Discussion

Using clinic- and neighborhood environment-based data from a large, prospective cohort of black and white young adults followed into middle adulthood, we estimated changes in BMI expected from changes in single as well as multiple elements of the neighborhood food retail and physical activity environments. Our findings suggest that increasing the density of supermarkets or commercial physical activity facilities was associated with small declines in BMI (0.09 to 0.22 kg/m^2^ between exams, approximately five years). Combined changes had more consistent and stronger estimated effects (up to a 0.31 kg/m^2^ reduction in BMI between exam periods). Isolated increases in neighborhood development intensity or neighborhood public physical activity facility density, or reductions in fast food restaurant or convenience store density were associated with increased or inconsistent changes in BMI.

### Advantages of examining neighborhood food retail and physical activity environments simultaneously

To our knowledge, our study is the first longitudinal study to investigate neighborhood environmental drivers of both sides of the energy balance equation (diet and physical activity). By simultaneously analyzing numerous aspects of neighborhood food retail environment and physical activity environment, we estimated independent effects of specific and combined food and activity environment features on BMI. As such, our findings shed light on mixed findings reported in the literature [50–53]. For example, findings demonstrating an association between higher frequency of supermarkets with lower BMI or obesity [3,14,21,54,55] could theoretically be driven by high development intensity (one component of walkability) in areas with higher supermarket availability. To the contrary, our simultaneous examination of supermarket density and development intensity suggests that higher density of supermarkets may be related to lower BMI, independent of development intensity. Likewise, a previous natural experiment suggested that addition of a single supermarket had no impact of diet [56], but it is possible that supermarket availability combined with other neighborhood factors may be important. We hypothesized that the food retail and physical activity environments interact in their relationships with BMI, but our study findings do not support this hypothesis.

We identified interactions between neighborhood poverty and neighborhood physical activity resources. Counterintuitive relationships between increased public physical activity facility density and increases in BMI were only observed in the presence of high neighborhood poverty. This finding emphasizes the importance of social context; that is, high neighborhood poverty may reflect perceived safety, social norms, or other aspects that may influence obesity-related behaviors. Interactions with neighborhood poverty may also reflect differential behavioral determinants among racial minorities and low-income groups who are more likely to live in neighborhoods with higher poverty levels. Among men, increases in commercial physical activity facilities predicted larger reductions in BMI in areas with high public physical activity facility density, suggesting that exposure to physical activity cues have cumulative effects on BMI. 

However, predicted reductions in BMI were only observed among men, and commercial facilities are more accessible to higher income individuals. Therefore, to prevent exacerbation of gender and income inequities, policies designed to increase commercial physical activities should incorporate complimentary strategies offering subsidies or reduced prices, and tailoring to specific needs of women. However, reasons for gender-specific associations in this and prior studies [10,57] are unknown. Unmeasured neighborhood factors such as safety, or individual factors such as household responsibilities [58] or dietary restraint [59] may contribute to obesity-related behavior more strongly in women compared to men. Elucidation of such mechanisms is needed to develop policy strategies that address barriers in both men and women.

To explore how built environment features may operate together, we tested pairwise interactions among a well-defined set of diverse neighborhood measures, accounting for differential relationships according to socioeconomic context and sex. Such inter-relationships among an expanded set of neighborhood measures could be further explored using pattern analysis techniques. Additionally, replication of the interactions found in the current study in other study populations and in race/ethnic and socioeconomic subgroups, and exploration of the theorized underlying mechanisms are critical next steps. 

### What is the role of diet and physical activity behaviors?

Further complexity is revealed by comparing our current findings with our previous examination of the presumed behavioral mediators in the relationship between the food retail and physical activity environments: diet [10] and physical activity [57]. In these previous studies, we found that greater neighborhood fast food restaurants density was associated with greater consumption of fast food [10], which was, in turn, associated with greater weight gain and obesity [60]; however, in the current study, fast food restaurant density was unrelated to BMI. Any impacts of fast food restaurant density on fast food consumption may not translate into BMI gains, perhaps due to competing effects of other neighborhood characteristics and obesity-related behaviors. In contrast, we previously found that neighborhood supermarket density was unrelated to individual-level diet quality, perhaps because supermarkets contain both healthy and unhealthy food options [10]. In the current study, relationships between greater supermarket density and lower BMI may reflect impacts of dietary behaviors that were unmeasured in our prior study. In both cases, findings may reflect residual confounding. On the physical activity side, we have reported mixed associations between street design (walkability) and higher levels of street-based physical activity [57], which is consistent with our current finding that neighborhood development intensity is unrelated to BMI. Our finding that greater density of neighborhood convenience stores was not associated with BMI is also consistent with mixed findings in other study populations [11,15,16,61–63]. Exploration of these behavioral pathways using mediation analysis and sophisticated modeling strategies and simultaneous examination of neighborhood, behavioral, and BMI data is an important next step.

### Opportunities and challenges of simultaneous neighborhood environment changes

While not feasible in many settings, the dramatic neighborhood changes simulated in this study are consistent with secular changes that have occurred over many decades and are readily applicable to the design of new neighborhoods such as New Urbanist communities [64,65] as well as to major neighborhood renewal projects [66]. Rigorous evaluation of major neighborhood renovation projects will improve understanding of the causal effects of neighborhood impacts on obesity.

In addition, the predicted reductions in BMI were small – up to 0.31 BMI units between exams – but may be important at the population level. Greater understanding of the most promising policy changes, contexts in which they are most effective, and the optimal magnitude of environmental changes may incur greater success in reducing BMI.

### Study limitations and strengths

The broad classifications of food retail environment and physical activity resources examined in our study may not reflect availability of specific foods or exercise facilities that more directly support healthy diets and physically active lifestyles [51], and do not reflect lower quality or other differences in resources located in socioeconomically disadvantaged areas [67]. We did not examine the full spectrum of neighborhood resources that drive diet and physical activity behavior such as non-traditional food outlets [68]. The commercial food and physical activity resource database may contain error in the number and location of resources and in classifications of restaurants, food stores, and physical activity resources examined in our study [69,70]; however such error is less pronounced in urban areas [71], which predominate in our study population, and is unlikely to vary systematically with BMI. Furthermore, Dun & Bradstreet is the only data source that provides comparable data across the U.S. and historically to 1992, so combining multiple data sources [70] was not possible. In the absence of high quality empirical data quantifying error in commercial resource databases historically and across diverse metropolitan areas, we did not apply a correction factor as performed in previous neighborhood studies [72]. Parcel-level data needed to measure land use mix [6–8], an important aspect of walkability, were not collected for this study. Additionally, while neighborhood boundaries based on individual perceptions[73] or transit patterns[74], or approaches that do not delineate spatial boundaries[75] may be valuable, we used a buffer-based neighborhood definition that is objective, delineated independent of behavior, and feasible in a large-scale longitudinal study. Finally, we had insufficient sample size to estimate race-specific effects in the presence of interactions by sex, neighborhood poverty, and built environment characteristics. Nonetheless, our data provided comparable, objective, and time-varying data for a large, diverse sample of young adults residing throughout the U.S. and followed into middle age. 

## Conclusions

In this large, diverse study population followed over 18 years, simulations of changes to single elements of the neighborhood physical activity and food retail environments suggest that increasing the density of neighborhood supermarkets and commercial physical activity facilities are promising policy targets for reducing and maintaining BMI. Findings from simulations combining changes to both supermarkets and commercial physical activity facilities suggests consistent inter-exam BMI reductions ranging from 0.23 to 0.31 kg/m^2^ within approximately 5 year periods in men, but no change in BMI in women. Variation in reduction in BMI according to the density of different types of neighborhood resources and in relation to neighborhood poverty suggests that neighborhood improvements should be tailored to specific neighborhood contexts. Identification of the most beneficial combinations of neighborhood improvements in varying contexts requires greater understanding of complex, interactive impacts across many aspects of the food retail, physical activity, and socioeconomic environments.

## Supporting Information

File S1 **Table S1** , Detailed food resource definitions based on 8-digit Standard Industrial Classification (SIC) codes. Table **S2**, Detailed physical activity resource definitions based on 8-digit Standard Industrial Classification (SIC) codes. Table **S3**, Detailed resource definitions for sedentary and food facilities that were only included in “total resources”, based on 8-digit Standard Industrial Classification (SIC) codes. Table **S4**, Factor loadings for development intensity^a^. Table **S5**, Model coefficients for fixed effects regression modeling of Body Mass Index (kg/m^2^) as a function of neighborhood food retail or physical activity environment measures. Table **S6**, Model coefficients for crude fixed effects regression modeling of Body Mass Index (kg/m^2^) as a function of neighborhood food retail or physical activity environment measures^a^. Table **S7**, Spearman correlation coefficients between neighborhood food retail or physical activity environment measures.
(DOCX)
