# Supplementary material for: The Neighborhood Energy Balance Equation: Does Neighborhood Food Retail Environment + Physical Activity Environment = Obesity? The CARDIA Study
Source: PLoS One. 2013 Dec 27;8(12):e85141. doi: 10.1371/journal.pone.0085141 (PMC3874030; doi:10.1371/journal.pone.0085141)
Supplement: File S1 — Table S1 , Detailed food resource definitions based on 8-digit Standard Industrial Classification (SIC) codes. Table S2, Detailed physical activity resource definitions based on 8-digit Standard Industrial Classification (SIC) codes. Table S3, Detailed resource definitions for sedentary and food facilities that were only included in “total resources”, based on 8-digit Standard Industrial Classification (SIC) codes. Table S4, Factor loadings for development intensitya. Table S5, Model coefficients for fixed effects regression modeling of Body Mass Index (kg/m2) as a function of neighborhood food retail or physical activity environment measures. Table S6, Model coefficients for crude fixed effects regression modeling of Body Mass Index (kg/m2) as a function of neighborhood food retail or physical activity environment measuresa. Table S7, Spearman correlation coefficients between neighborhood food retail or physical activity environment measures. (DOCX) [file pone.0085141.s001.docx]

**SUPPORTING INFORMATION**

**Table S1.** Detailed food resource definitions based on 8-digit Standard Industrial Classification (SIC) codes

| **Resource Type** | **SIC** | **SIC definition** |
| --- | --- | --- |
| Fast food restaurants | 58120307 | Fast-food restaurant, chain |
|  | 58120601 | Pizzeria, chain |
| Supermarkets | 54110100 | Supermarkets |
|  | 54110101 | Supermarkets, chain |
|  | 54110102 | Supermarkets, >100,000 feet^2^ (hypermarket) |
|  | 54110103 | Supermarkets, independent |
|  | 54110104 | Supermarkets, 55,000 - 65,000 feet^2^ (superstore) |
|  | 54110105 | Supermarkets, 66,000 - 99,000 feet^2^ |
| Convenience stores | 53310000 | Variety stores |
|  | 54110200 | Convenience stores |
|  | 54110201 | Convenience stores, chain |
|  | 54110202 | Convenience stores, independent |
|  | 55410000 | Gasoline service stations |
|  | 55419900 | Gasoline service stations, nec |
|  | 55419901 | Filling stations, gasoline |
|  |  |  |

**Table S2.** Detailed physical activity resource definitions based on 8-digit Standard Industrial Classification (SIC) codes

| **Resource Type** | **SIC** | **SIC Definition** |
| --- | --- | --- |
| Commercial physical activity facilities^a^ | 79110000 | Dance studios, schools, and halls |
|  | 79110200 | Dance instructor and school services |
|  | 79110201 | Childrens' dancing school |
|  | 79110202 | Dance instructor |
|  | 79110203 | Dance studio and school |
|  | 79330000 | Bowling centers |
|  | 79339900 | Bowling centers, nec |
|  | 79339901 | Candle pin center |
|  | 79339902 | Duck pin center |
|  | 79339903 | Ten pin center |
|  | 79910000 | Physical fitness facilities |
|  | 79910100 | Physical fitness clubs with training equipment |
|  | 79910101 | Athletic club and gymnasiums, membership |
|  | 79910102 | Health club |
|  | 79910300 | Exercise facilities |
|  | 79910301 | Aerobic dance and exercise classes |
|  | 79910302 | Exercise salon |
|  | 79920000 | Public golf courses |
|  | 79970000 | Membership sports and recreation clubs |
|  | 79970100 | Ice sports |
|  | 79970102 | Hockey club, except professional and semi-professional |
|  | 79970200 | Boating and swimming clubs |
|  | 79970201 | Beach club, membership |
|  | 79970202 | Boating club, membership |
|  | 79970203 | Swimming club, membership |
|  | 79970302 | Hunting club, membership |
|  | 79970401 | Baseball club, except professional and semi-professional |
|  | 79970402 | Football club, except professional and semi-professional |
|  | 79970404 | Soccer club, except professional and semi-professional |
|  | 79970500 | Indoor/outdoor court clubs |
|  | 79970501 | Handball club, membership |
|  | 79970502 | Racquetball club, membership |
|  | 79970503 | Squash club, membership |
|  | 79970504 | Tennis club, membership |
|  | 79979904 | Country club, membership |
|  | 79979906 | Golf club, membership |
|  | 79979908 | Riding club, membership |
|  | 79990101 | Tennis club, non-membership |
|  | 79990103 | Tennis professional |
|  | 79990200 | Golf services and professionals |
|  | 79990202 | Golf driving range |
|  | 79990203 | Golf professionals |
|  | 79990204 | Golf, pitch-n-putt |
|  | 79990205 | Miniature golf course operation |
|  | 79990300 | Indoor court clubs |
|  | 79990301 | Handball courts, non-membership |
|  | 79990302 | Racquetball club, non-membership |
|  | 79990303 | Squash club, non-membership |
|  | 79990501 | Bicycle rental |
|  | 79990602 | Ice skating rink operation |
|  | 79990603 | Roller skating rink operation |
|  | 79991102 | Baseball instruction school |
|  | 79991103 | Basketball instruction school |
|  | 79991104 | Bowling instruction |
|  | 79991109 | Gymnastic instruction, non-membership |
|  | 79991110 | Hockey instruction school |
|  | 79991111 | Judo instruction |
|  | 79991112 | Karate instruction |
|  | 79991113 | Martial arts school, nec |
|  | 79991115 | Sailing instruction |
|  | 79991118 | Skating instruction, ice or roller |
|  | 79991119 | Ski instruction |
|  | 79991120 | Sports instruction, schools and camps |
|  | 79991121 | Surfing instruction |
|  | 79991122 | Swimming instruction |
|  | 79991123 | Yoga instruction |
|  | 79991127 | Physical fitness instruction |
|  | 79991200 | Riding and rodeo services |
|  | 79991201 | Riding academy and school |
|  | 79991202 | Riding stable |
|  | 79991205 | Saddlehorse rental |
|  | 79991407 | Lifeguard service |
|  | 79991409 | Rowboat and canoe rental |
|  | 79991410 | Sailboard rental |
|  | 79991411 | Surfing equipment rental |
|  | 79991512 | Waterslide operation |
|  | 79991513 | Wave pool operation |
|  | 79991515 | Zoological garden, commercial |
|  | 79991602 | Rafting tours |
|  | 79991604 | Trail guide |
|  | 79999907 | Outfitters, recreation |
| Public physical activity facilities^a^ | 79990102 | Tennis courts, outdoor/indoor: non-membership |
|  | 79991402 | Bathing beach, non-membership |
|  | 79991412 | Swimming pool, non-membership |
|  | 79999910 | Recreation center |
|  | 79999912 | Recreation services |
|  | 83220205 | Youth center |
|  | 83220601 | Community center |

**Table S3.** Detailed resource definitions for sedentary and food facilities that were only included in “total resources”, based on 8-digit Standard Industrial Classification (SIC) codes

| **Resource Type** | **SIC** | **SIC Definition** |
| --- | --- | --- |
| Facilities supporting sedentary activities | 78320000 | Motion picture theaters, except drive-in |
|  | 78329900 | Motion picture theaters, except drive-in, nec |
|  | 78330000 | Drive-in motion picture theaters |
|  | 79930301 | Amusement arcade |
|  | 79930302 | Video game arcade |
| Other grocery stores |  |  |
|  | 53999903 | Country general stores |
|  | 54110000 | Grocery stores |
|  | 54119900 | Grocery stores, nec |
|  | 54119901 | Cooperative food stores |
|  | 54119903 | Frozen food and freezer plans, except meat |
|  | 54119904 | Grocery stores, chain |
|  | 54119905 | Grocery stores, independent |
| Markets |  |  |
|  | 54210000 | Meat and fish markets |
|  | 54210100 | Fish and seafood markets |
|  | 54210101 | Fish markets |
|  | 54210102 | Seafood markets |
|  | 54210201 | Food and freezer plans, meat |
|  | 54210202 | Freezer provisioners, meat |
|  | 54210200 | Meat markets, including freezer provisioners |
|  | 54310000 | Fruit and vegetable markets |
|  | 54319900 | Fruit and vegetable markets, nec |
|  | 54319901 | Fruit stands or markets |
|  | 54319902 | Vegetable stands or markets |
| Other stores |  |  |
|  | 54119901 | Cooperative food stores |
|  | 54410000 | Candy, nut, and confectionery stores |
|  | 54419900 | Candy, nut, and confectionery stores, nec |
|  | 54419901 | Candy |
|  | 54419902 | Confectionery |
|  | 54419903 | Confectionery produced for direct sale on the premises |
|  | 54419904 | Nuts |
|  | 54510000 | Dairy products stores |
|  | 54519900 | Dairy products stores, nec |
|  | 54519901 | Butter |
|  | 54519902 | Cheese |
|  | 54519903 | Ice cream (packaged) |
|  | 54519904 | Milk |
|  | 54610000 | Retail bakeries |
|  | 54619900 | Retail bakeries, nec |
|  | 54619901 | Bagels |
|  | 54619902 | Bread |
|  | 54619903 | Cakes |
|  | 54619904 | Cookies |
|  | 54619905 | Doughnuts |
|  | 54619906 | Pastries |
|  | 54619907 | Pies |
|  | 54619908 | Pretzels |
|  | 54990000 | Miscellaneous food stores |
|  | 54990100 | Health and dietetic food stores |
|  | 54990101 | Dietetic foods |
|  | 54990102 | Health foods |
|  | 54990200 | Beverage stores |
|  | 54990201 | Coffee |
|  | 54990202 | Juices, fruit or vegetable |
|  | 54990204 | Tea |
|  | 54999900 | Miscellaneous food stores, nec |
|  | 54999902 | Eggs and poultry |
|  | 54999904 | Gourmet food stores |
|  | 86999907 | Food co-operative |
| Warehouse stores |  |  |
|  | 53999906 | Warehouse club stores |
| Non-fast food restaurants |  |  |
|  | 54119902 | Delicatessen stores |
|  | 58120000 | Eating places |
|  | 58120100 | Ethnic food restaurants |
|  | 58120101 | American restaurant |
|  | 58120102 | Cajun restaurant |
|  | 58120103 | Chinese restaurant |
|  | 58120104 | French restaurant |
|  | 58120105 | German restaurant |
|  | 58120106 | Greek restaurant |
|  | 58120107 | Indian/Pakistan restaurant |
|  | 58120108 | Italian restaurant |
|  | 58120109 | Japanese restaurant |
|  | 58120110 | Korean restaurant |
|  | 58120111 | Lebanese restaurant |
|  | 58120112 | Mexican restaurant |
|  | 58120113 | Spanish restaurant |
|  | 58120114 | Sushi bar |
|  | 58120115 | Thai restaurant |
|  | 58120116 | Vietnamese restaurant |
|  | 58120117 | Pakistani restaurant |
|  | 58120200 | Ice cream, soft drink and soda fountain stands |
|  | 58120202 | Frozen yogurt stand |
|  | 58120203 | Ice cream stands or dairy bars |
|  | 58120204 | Snow cone stand |
|  | 58120205 | Soda fountain |
|  | 58120206 | Soft drink stand |
|  | 58120300 | Fast food restaurants and stands |
|  | 58120301 | Box lunch stand |
|  | 58120302 | Carry-out only (except pizza) restaurant |
|  | 58120303 | Chili stand |
|  | 58120304 | Coffee shop |
|  | 58120305 | Delicatessen (eating places) |
|  | 58120306 | Drive-in restaurant |
|  | 58120308 | Fast-food restaurant, independent |
|  | 58120309 | Food bars |
|  | 58120310 | Grills (eating places) |
|  | 58120311 | Hamburger stand |
|  | 58120312 | Hot dog stand |
|  | 58120313 | Sandwiches and submarines shop |
|  | 58120314 | Snack bar |
|  | 58120315 | Snack shop |
|  | 58120400 | Lunchrooms and cafeterias |
|  | 58120401 | Automat (eating places) |
|  | 58120402 | Cafeteria |
|  | 58120403 | Luncheonette |
|  | 58120404 | Lunchroom |
|  | 58120405 | Restaurant, lunch counter |
|  | 58120500 | Family restaurants |
|  | 58120501 | Restaurant, family: chain |
|  | 58120502 | Restaurant, family: independent |
|  | 58120600 | Pizza restaurants |
|  | 58120602 | Pizzeria, independent |
|  | 58120700 | Seafood restaurants |
|  | 58120701 | Oyster bar |
|  | 58120702 | Seafood shack |
|  | 58120800 | Steak and barbecue restaurants |
|  | 58120801 | Barbecue restaurant |
|  | 58120802 | Steak restaurant |
|  | 58129900 | Eating places, nec |
|  | 58129901 | Buffet (eating places) |
|  | 58129902 | Cafe |
|  | 58129904 | Chicken restaurant |
|  | 58129905 | Commissary restaurant |
|  | 58129907 | Diner |
|  | 58129908 | Dinner theater |

**Table S4**. Factor loadings for development intensity^a^

| Variable | Loading | |
| --- | --- | --- |
|  | 1km | 3km |
| Population density | 0.796 | 0.889 |
| Road density | 0.131 | 0.595 |
| Resource density | 0.792 | 0.793 |

^a^Derived from exploratory factor analysis (EFA) of neighborhood characteristics within 1km or 3km Euclidean buffers using the principal factors estimator. Based on the Kaiser Criterion (Eigenvalue>1) and scree plots, the three measures represented a single construct (factor).

**Table S5**. Model coefficients for fixed effects regression modeling of Body Mass Index (kg/m^2^) as a function of neighborhood food retail or physical activity environment measures

|  | Primary analysis^a^ | | | Alternate analysis^b^ | |  |
| --- | --- | --- | --- | --- | --- | --- |
|  | Coefficient  (95% CI) | p-value | Coefficient  (95% CI) | | p-value | |
| Fast food restaurants^c^ | -0.04 (-0.18, 0.09) | 0.522 | -0.05 (-0.18, 0.09) | | 0.510 | |
| Supermarkets^d^ | -0.09 (-0.15, -0.02) | 0.012 | -0.08 (-0.15, -0.02) | | 0.015 | |
| Convenience stores^c^ | 0.04 (-0.08, 0.16) | 0.549 | 0.04 (-0.08, 0.16) | | 0.549 | |
| Development intensity^e^ | -0.03 (-0.11, 0.05) | 0.502 | -0.05 (-0.13, 0.04) | | 0.284 | |
| Neighborhood poverty^f^ | -1.57 (-2.40, -0.74) | 0.000 | -1.55 (-2.38, -0.72) | | 0.000 | |
| Public physical activity facilities^c^ | -0.01 (-0.45, 0.42) | 0.953 | 0.00 (-0.44, 0.43) | | 0.999 | |
| Public facilities*Neighborhood poverty | 1.58 (0.44, 2.72) | 0.006 | 1.57 (0.43, 2.71) | | 0.007 | |
| Public facilities*Commercial facilities | -0.15 (-0.33, 0.03) | 0.096 | -0.15 (-0.33, 0.02) | | 0.089 | |
| Commercial physical activity facilities^c^ | -0.18 (-0.38, 0.02) | 0.073 | -0.18 (-0.38, 0.01) | | 0.068 | |
| Commercial facilities*Female^g^ | 0.16 (-0.04, 0.35) | 0.118 | 0.16 (-0.04, 0.35) | | 0.118 | |
| Commercial facilities* Neighborhood Poverty | 0.04 (-0.62, 0.71) | 0.899 | 0.06 (-0.61, 0.72) | | 0.871 | |
| Commercial facilities*Female* Neighborhood Poverty | 0.53 (-0.07, 1.12) | 0.082 | 0.53 (-0.06, 1.13) | | 0.080 | |
| BMI at previous exam | 0.24 (0.22, 0.26) | 0.000 | 0.24 (0.22, 0.26) | | 0.000 | |
| Household income (in $10,000’s) | 0.06 (-0.07, 0.20) | 0.376 | 0.06 (-0.07, 0.20) | | 0.366 | |
| Age | 0.14 (0.13, 0.15) | 0.000 | 0.14 (0.13, 0.15) | | 0.000 | |
| Current smoker | 0.07 (-0.16, 0.29) | 0.568 | 0.07 (-0.16, 0.29) | | 0.559 | |
| Currently married | -0.07 (-0.24, 0.09) | 0.362 | -0.08 (-0.24, 0.08) | | 0.351 | |
| Any children in household | 0.04 (-0.10, 0.19) | 0.554 | 0.04 (-0.10, 0.19) | | 0.559 | |
| (constant) | 21.6 (20.9, 22.3) | 0.000 | 21.6 (20.9, 22.3) | | 0.000 | |

^a^Estimated using fixed effects linear regression modeling Body Mass Index (BMI, kg/m^2^) as a function of fast food restaurant, convenience store, supermarket, commercial physical activity facility, public physical activity facility density within 3km buffers and development intensity within 1km buffers (Euclidean buffers around each respondent’s residential location), and percent of persons below 150% of federal poverty level; Coronary Artery Risk Development in Young Adults (CARDIA) Study (1992-2011); n=12,921 person-exam observations representing 4,092 individuals. The fixed effects model is adjusted for time-varying income, age, marital status, children in household, and significant (p<0.10) interactions between neighborhood measure and gender, and significant pairwise interactions among neighborhood measures; race, education, and study center are time invariant and therefore omitted from fixed effects models.

^b^Alternate analysis measures development intensity within 3km, rather than 1km Euclidean buffer

^c^Resource density (counts per 10,000 population) within 3km Euclidean buffer; natural-log transformed

^d^Resource density (counts per 100,000 population) within 3km Euclidean buffer; natural-log transformed

^e^ Development intensity score constructed from population density, road density, and total resource (all food, physical activity, and inactivity facilities) within 1km or 3km Euclidean buffer using Exploratory Factor Analysis

^f^ Proportion households <150% of poverty within census tract

^g^In fixed effects models, main effect is not estimated for time-constant variables

**Table S6**. Model coefficients for crude fixed effects regression modeling of Body Mass Index (kg/m^2^) as a function of neighborhood food retail or physical activity environment measures^a^

|  | Crude model 1^b^ | | Crude model 2^b^ | | Crude model 3^b^ | |
| --- | --- | --- | --- | --- | --- | --- |
|  | Coefficient  (95% CI) | p-value | Coefficient  (95% CI) | p-value | Coefficient  (95% CI) | p-value |
| Fast food restaurants^c^ | 0.84 (0.72, 0.96) | 0.000 | 0.37 (0.22, 0.52) | 0.000 | 0.35 (0.20, 0.50) | 0.000 |
| Supermarkets^d^ | 0.19 (0.12, 0.25) | 0.000 | 0.00 (-0.07, 0.07) | 0.972 | 0.00 (-0.07, 0.08) | 0.956 |
| Convenience stores^c^ | 0.07 (-0.05, 0.19) | 0.244 | -0.40 (-0.53, -0.27) | 0.000 | -0.41 (-0.54, -0.28) | 0.000 |
| Development intensity^e^ | -0.44 (-0.53, -0.36) | 0.000 | -0.35 (-0.44, -0.26) | 0.000 | -0.33 (-0.42, -0.24) | 0.000 |
| Neighborhood poverty^f^ | -2.34 (-2.82, -1.85) | 0.000 | -0.94 (-1.44, -0.43) | 0.000 | -2.35 (-3.26, -1.44) | 0.000 |
| Public physical activity facilities^c^ | 0.82 (0.63, 1.00) | 0.000 | 0.54 (0.34, 0.73) | 0.000 | -0.03 (-0.50, 0.45) | 0.906 |
| Public facilities*Neighborhood poverty | NA^b^ |  | NA^b^ |  | 3.20 (1.96, 4.43) | 0.000 |
| Public facilities*Commercial facilities | NA^b^ |  | NA^b^ |  | -0.03 (-0.22, 0.16) | 0.764 |
| Commercial physical activity facilities^c^ | 1.03 (0.94, 1.13) | 0.000 | 0.85 (0.73, 0.96) | 0.000 | 0.74 (0.53, 0.95) | 0.000 |
| Commercial facilities*Female^g^ | NA^b^ |  | NA^b^ |  | 0.21 (-0.01, 0.42) | 0.056 |
| Commercial facilities* Neighborhood Poverty | NA^b^ |  | NA^b^ |  | -0.63 (-1.36, 0.09) | 0.088 |
| Commercial facilities*Female* Neighborhood Poverty | NA^b^ |  | NA^b^ |  | 0.88 (0.23, 1.52) | 0.008 |
| (constant) | Varies^h^ |  | 28.04 (27.79, 28.28) | 0.000 | 28.32 (28.01, 28.64) | 0.000 |

^a^Estimated using fixed effects linear regression modeling Body Mass Index (BMI, kg/m^2^) as a function of fast food restaurant, convenience store, supermarket, commercial physical activity facility, and public physical activity facility density within 3km buffers and development intensity within 1km buffers (Euclidean buffers around each respondent’s residential location), and percent of persons below 150% of federal poverty level; Coronary Artery Risk Development in Young Adults (CARDIA) Study (1992-2011); n=12,921 person-exam observations representing 4,092 individuals.

^b^Crude model 1 models BMI as a function of a single neighborhood variable. Crude model 2 models BMI as a function of all seven neighborhood variables. Crude model 3 models BMI as a function of all seven neighborhood variables and significant (p<0.10) interactions between neighborhood measure and gender, and significant pairwise interactions among neighborhood measures.

^c^Resource density (counts per 10,000 population) within 3km Euclidean buffer; natural-log transformed

^d^Resource density (counts per 100,000 population) within 3km Euclidean buffer; natural-log transformed

^e^ Development intensity score constructed from population density, road density, and total resource (all food, physical activity, and inactivity facilities) within 1km Euclidean buffer using Exploratory Factor Analysis

^f^ Proportion households <150% of poverty within census tract

^g^In fixed effects models, main effect is not estimated for time-constant variables

^h^Constant varies across seven crude models, each modeling BMI as a function of a single neighborhood variable: Fast food restaurants: 28.11 (28.00, 28.22); Supermarkets: 28.50 (28.39, 28.62); Convenience stores: 28.68 (28.47, 28.89); Development Intensity: 28.80 (28.75, 28.84); Neighborhood poverty: 29.30 (29.19, 29.41); Public facilities: 28.48 (28.40, 28.57); Commercial facilities: 27.43 (27.29, 27.56)

**Table S7**. Spearman correlation coefficients between neighborhood food retail or physical activity environment measures

|  | Fast food | Supermarkets | Convenience | Commercial | Public | Development | Poverty |
| --- | --- | --- | --- | --- | --- | --- | --- |
| Fast food restaurants^a^ (fast food) | 1 |  |  |  |  |  |  |
| Supermarkets^b^ | 0.41 | 1 |  |  |  |  |  |
| Convenience stores^a^ (convenience) | 0.45 | 0.29 | 1 |  |  |  |  |
| Commercial physical activity facilities^a^ | 0.43 | 0.23 | 0.19 | 1 |  |  |  |
| Public physical activity facilities (public) | 0.20 | 0.08 | 0.06 | 0.24 | 1 |  |  |
| Development intensity^c^ (development) | -0.04 | 0.03 | -0.17 | -0.05 | 0.46 | 1 |  |
| Neighborhood poverty^d^ (poverty) | -0.04 | 0.03 | 0.16 | -0.38 | 0.19 | 0.37 | 1 |

Coronary Artery Risk Development in Young Adults (CARDIA) Study (1992-2011); 12,921 person-exam observations representing 4,092 individuals

^a^Resource density (counts per 10,000 population) within 3km Euclidean buffer

^b^Resource density (counts per 100,000 population) within 3km Euclidean buffer

^c^Development intensity score constructed from population density, road density, and total resource (all food, physical activity, and inactivity facilities) using Exploratory Factor Analysis

^d^Proportion households <150% of poverty within census tract
